# Health Hints for Travelers

**Published:** 1884-08

**Authors:** 


HEALTH HINTS FOR TRAVELERS, By JOHN C. SUNDBERG, M.D., D. G. BRINTON, Philadelphia, Publisher, 1884. The author, in his preface, says "the object of this book is to teach the traveler to keep himself well, and to help others who are less fortunate." It is well written and will prove both interesting and useful to the traveler.

